# Recombinant BCG-Prime and DNA-Boost Immunization Confers Mice with Enhanced Protection against *Mycobacterium kansasii*

**DOI:** 10.3390/vaccines9111260

**Published:** 2021-11-01

**Authors:** Shihoko Komine-Aizawa, Satoru Mizuno, Kazuhiro Matsuo, Takahiro Namiki, Satoshi Hayakawa, Mitsuo Honda

**Affiliations:** 1 Department of Pathology and Microbiology, Division of Microbiology, Nihon University School of Medicine, Tokyo 173-8610, Japan; hayakawa.satoshi@nihon-u.ac.jp; 2 Japan BCG Laboratory, Tokyo 204-0022, Japan; s-mizuno@bcg.gr.jp (S.M.); matsuo@bcg.gr.jp (K.M.); 3 Nihon University School of Medicine, Tokyo 173-8610, Japan; meta16076@g.nihon-u.ac.jp

**Keywords:** *Mycobacterium kansasii*, CD8^+^ T Cells, CD4^+^ T Cells, recombinant BCG

## Abstract

The incidence of infections with nontuberculous mycobacteria (NTM) has been increasing worldwide. The emergence of multidrug-resistant NTM is a serious clinical concern, and a vaccine for NTM has not yet been developed. We previously developed a new recombinant Bacillus Calmette–Guérin (rBCG) vaccine encoding the antigen 85B (Ag85B) protein of *Mycobacterium kansasii*—termed rBCG-Mkan85B—which was used together with a booster immunization with plasmid DNA expressing the same *M. kansasii* Ag85B gene (DNA-Mkan85B). We reported that rBCG-Mkan85B/DNA-Mkan85B prime–boost immunization elicited various NTM strain-specific CD4^+^ and CD8^+^ T cells and induced *Mycobacterium* *tuberculosis*-specific immunity. In this study, to investigate the protective effect against *M. kansasii* infection, we challenged mice vaccinated with a rBCG-Mkan85B or rBCG-Mkan85B/DNA-Mkan85B prime–boost strategy with virulent *M. kansasii*. Although BCG and rBCG-Mkan85B immunization each suppressed the growth of *M. kansasii* in the mouse lungs, the rBCG-Mkan85B/DNA-Mkan85B prime–boost vaccination reduced the bacterial burden more significantly. Moreover, the rBCG-Mkan85B/DNA-Mkan85B prime–boost vaccination induced antigen-specific CD4^+^ and CD8^+^ T cells. Our data suggest that rBCG-Mkan85B/DNA-Mkan85B prime–boost vaccination effectively enhances antigen-specific T cells. Our novel rBCG could be a potential alternative to clinical BCG for preventing various NTM infections.

## 1. Introduction

Nontuberculous mycobacteria (NTM) are mycobacterium species other than the *Mycobacterium tuberculosis* complex and *Mycobacterium leprae,* such as *Mycobacterium avium, Mycobacterium intracellulare,* and *Mycobacterium kansasii*. Recently, the incidence of NTM infection has been increasing worldwide [1,2,3]. Although NTM are considered not to cause airborne infection, NTM can cause pulmonary and extrapulmonary diseases in immunocompromised and even nonimmunocompromised individuals. Moreover, NTM infections have been reported to be resistant to treatment [4,5,6,7,8]. Recently, novel therapeutics against NTM have been developed [7,8], but effective prevention by vaccination is so far unknown.

Since NTM pathogens are intracellular, cell-mediated immunity plays a major role in protecting against NTM. Previous reports have indicated that protective immunity against tuberculosis (TB) and NTM infection might overlap [9]. Epidemiological data suggest that latent TB infection decreases the risk of NTM diseases [9], and a systematic review found that an increased proportion of NTM diseases were related to coincidental decreases in TB [10]. In addition, several reports have indicated that Bacillus Calmette–Guérin (BCG)—which is the only available vaccine for TB—can induce protective immunity against NTM infection [11,12].

Previously, we developed a new recombinant BCG (rBCG) vaccine encoding the antigen 85B (Ag85B) protein of *M. kansasii*—termed rBCG-Mkan85B—which we used with a booster vaccination with plasmid DNA expressing the same *M. kansasii* Ag85B gene (DNA-Mkan85B) [13]. The Ag85 complex, which consists of three abundantly secreted proteins (Ag85A, Ag85B, and Ag85C), exhibits cell wall mycolyltransferase activity and plays an essential role in mycobacterial pathogenesis [14]. The Ag85 proteins have been reported to induce a strong cellular immune response against mycobacterium species such as *M. tuberculosis* and *M. leprae* [15,16]. We reported that rBCG-Mkan85B expressed Ag85B at levels 9.3-fold higher than parental BCG [13]. We also showed that the rBCG-Mkan85B/DNA-Mkan85B prime–boost scheme could elicit antigen-specific polyfunctional CD4^+^ T cells and CD8^+^ T cells [13]. Moreover, we identified two MHC-I (H2-K^d^)-restricted epitopes that induced cross-reactive responses to *M. tuberculosis* and other related mycobacteria, including *M. kansasii*, in both BALB/c (H2^d^) mice and CB6F1 (H2^b/d^) mice [13]. The H2-K^d^-restricted peptide epitopes elicited polyfunctional CD8^+^ T cell responses that were also highly cross-reactive with those of other proteins in the Ag85 complex [13]. Therefore, to confirm the protective effect of rBCG-Mkan85B/DNA-Mkan85B prime–boost vaccination against *M. kansasii*, we challenged rBCG-Mkan85B/DNA-Mkan85B-prime–boost-vaccinated CB6F1 mice with *M.* *kansasii* and evaluated the protective effect and cellular immunity in this study.

## 2. Materials and Methods

### 2.1. Animals

Specific pathogen-free female CB6F1 mice, aged between 6 and 8 weeks, were purchased from Japan SLC Inc. (Shizuoka, Japan). All animal studies were carried out under institutional guidelines approved by the Nihon University Animal Care and Use Committee (AP19MED029-3), the institutional committee for gene recombination experiments (2018MED21), and biorisk management and control (30-10-4). The institutional animal experimental guidelines are in accordance with the ILAR Guide. Mice were allowed free access to sterile water and standard mouse food, and their physiological conditions were assessed every few days.

### 2.2. BCG and rBCG-Mkan85B Culture

We used the previously prepared rBCG-Mkan85B strain and DNA-Mkan85B plasmid [13] in this study.

BCG was cultured in Middlebrook 7H9 broth (Difco) supplemented with albumin dextrose complex (ADC) enrichment (Difco) and 0.05% Tween 80 at 37 °C. rBCG-Mkan85B was cultured in Middlebrook 7H9 broth (Difco) supplemented with ADC enrichment (Difco), 0.05% Tween 80, and 10 mg/mL kanamycin at 37 °C. Bacterial culture density was monitored by measuring the absorbance at 470 nm and 600 nm.

### 2.3. Immunization

Mice were immunized with the BCG vaccine or rBCG-Mkan85B at a concentration of 4 × 10^6^ CFU or 0.1 mg of bacilli intradermally (i.d.), and 100 µg of plasmid DNA in saline intramuscularly (i.m.) three times [17] (Figure S1A,B). We used three to four mice per group.

### 2.4. Bacterial Infection

*M. kansasii* Hauduroy 1955 was obtained from the RIKEN Bio Resource Center under a material transfer agreement (MTA). The bacteriological experiment was carried out under institutional guidelines approved by Nihon University biorisk management and control (30-10-4). *M. kansasii* was grown in Middlebrook 7H9 broth (Difco) supplemented with ADC enrichment (Difco) with 0.05% Tween 80 at 37 °C and harvested during the exponential growth stage. Infection of mice with *M. kansasii* was conducted intratracheally. Mice were anaesthetized with isoflurane and inoculated with 50 µL of a bacterial suspension containing 1 to 10 × 10^4^ CFU of *M. kansasii* using a yellow pipet tip.

### 2.5. Infection Assay

At 6 weeks after infection, the mice were sacrificed, and the numbers of viable bacilli in their lungs were estimated. Lung tissues were homogenized with sterilized water. Tenfold serial dilutions of homogenate were inoculated onto duplicate 7H10-OADC agar plates (Difco) to determine bacterial loads. Colonies were counted after incubation for 3 weeks at 37 °C.

### 2.6. Histopathology

Fractioned lung samples from each mouse were fixed with 10% neutral-buffered formalin and embedded in paraffin wax. The sections from these tissues were 4 mm thick and stained with hematoxylin and eosin (H&E) or with the Ziehl-Neelsen stain for acid-fast bacilli.

### 2.7. Polychromatic Flow Cytometry for Intracellular Cytokine Production

Splenocytes and immune cells isolated from the lung were stimulated with a 9-mer Ag85B CD8 epitope peptide (YYQSGLSIV) (Pep8) [13], 15-mer Ag85B CD4^+^ epitope peptide (peptide-25: FQDAYNAAGGHNAVF) [18], or tuberculin PPD (PPD; 2 µg/mL) and stained for cell-surface or intracellular cytokines such as IFN-γ IL-2 and TNF, as described previously [13,17]. PPD was kindly supplied by the Japan BCG Laboratory. The Pep8 and peptide 25 were modified to match the amino acid sequence of *M. kansasii*. A seven-color flow cytometry panel was used to simultaneously analyze multiple cytokines at the single-cell level. The gating strategy used to identify cytokine-producing CD8^+^ and CD4^+^ T cells in mouse splenocytes is shown in Figure S2. Following in vitro stimulation for 6 h, cells were incubated with LIVE/DEAD Fixable Dead Cell Stains (Thermo Fisher Scientific) to identify dead cells, followed by surface staining with the antibodies APC-conjugated anti-CD3, PerCP-Cy5.5-conjugated anti-CD8 (BioLegend), and PE-Cy7-conjugated anti-CD4 (BD). The cells were then fixed and permeabilized using BD Cytofix/Cytoperm (BD) and stained for IFN-γ (PE), IL-2 (APC-Cy7), and TNF (Alexa Fluor 488) (BioLegend). Polyfunctional cells were defined as those making two or more cytokines using Boolean combinations [13]. FACS analysis was performed using a FACSVerse (BD) with FlowJo (BD) [13,17].

### 2.8. Data Analysis and Statistics

All comparisons among recombinant and control groups and among immunization groups were conducted using one-way ANOVA tests with the Tukey-Kramer test using the JMP program (SAS Institute) and R version 4.0.3 (R core team 2020). Data are expressed as the mean ± SD.

## 3. Results

### 3.1. BCG or rBCG-Mkan85B Vaccination Protects against M. kansasii Infection

According to previous epidemiological reports, BCG vaccination can reduce the risk of NTM infection in humans [19,20,21]. In a mouse model, BCG vaccination was reported to induce cross-protective immunity against *M. avium* and *M. abscessus* [11]. Therefore, we first examined the protective efficacy of BCG and rBCG-Mkan85B against *M.* *kansasii* infection.

**Figure 1:**
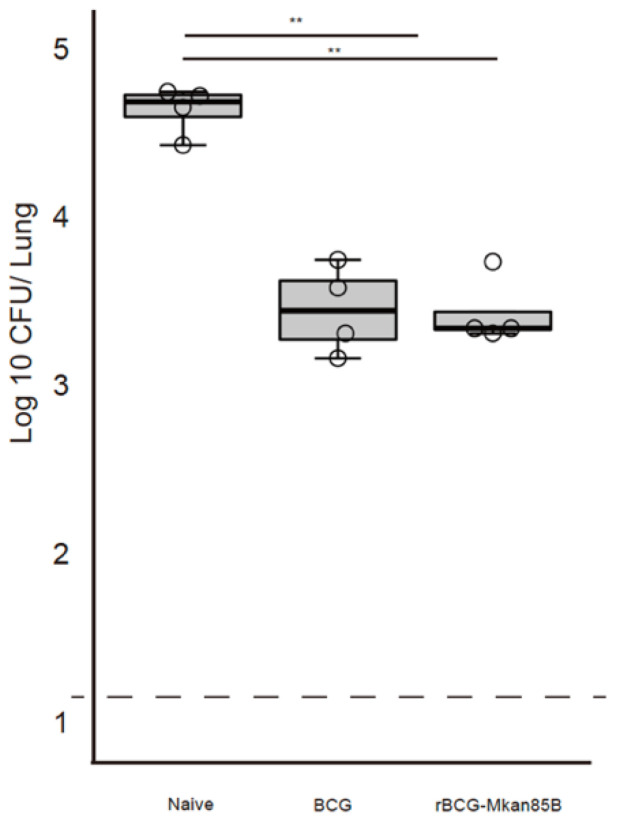
BCG or rBCG-Mkan85B vaccination protects against *M.* *kansasii* infection. The CB6F1 (H2b/d) mice were either left unimmunized or immunized with BCG or rBCG-Mkan85B for 6 weeks, followed by intratracheal infection with a virulent *M.* *kansasii* strain. The mice were sacrificed at 6 weeks after *M. kansasii* infection. The immunization schedule is shown Figure S1A. The number of *M.* *kansasii* in individual mouse lungs was analyzed using the colony assay method. The data represent two independent experiments with three to four mice per group. The detection limit for bacilli in the tissue homogenate was 15 CFU. The error bars represent the SD. ** *p* < 0.01.

**Figure 2:**
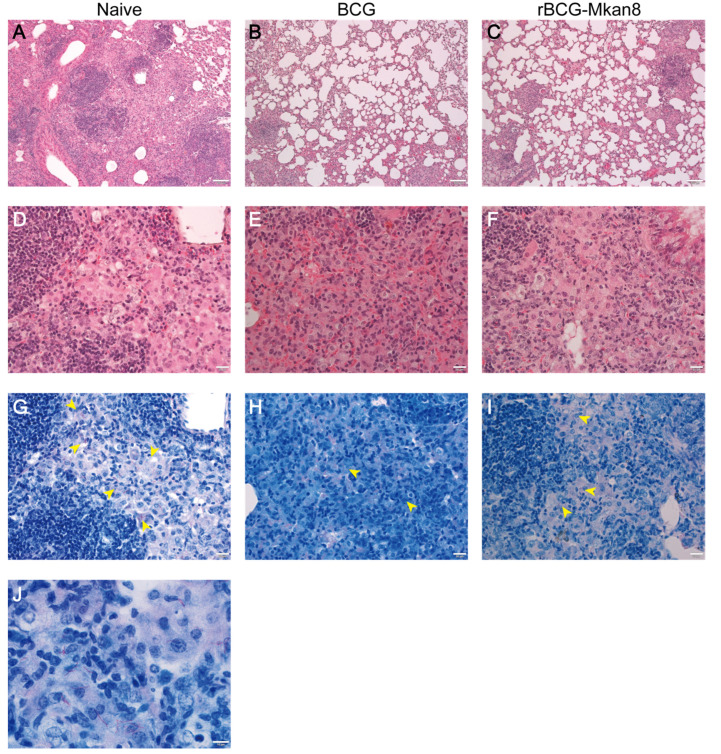
Histopathology of lungs from *M. kansasii*-infected mice. Representative pathological observations of the lungs of the unvaccinated (**A**,**D**,**G**,**J**), BCG-vaccinated (**B**,**E**,**H**), and rBCG-Mkan85B-vaccinated (**C**,**F**,**I**) mice 6 weeks after *M.* *kansasii* infection are shown. First-row panels, 100× of HE-stained specimens; second-row panels, 400× of HE-stained specimens; third-row panels, 400× of Ziehl-Neelsen-stained specimens; (panel **J**), 1000× of Ziehl-Neelsen-stained specimens. The yellow arrowheads indicate the acid-fast bacilli. Bars: 100 μm (first-row panels), 20 μm (second- and third-row panels), and 10 μm (panel **J**).

The CB6F1 (H2b/d) mice were either left unimmunized or immunized with BCG or rBCG-Mkan85B for 6 weeks, followed by a nasal exposure to a virulent *M.* *kansasii* strain for infection for another 6 weeks. The immunization schedule is shown in Figure S1A. Unimmunized mice produced 4.5 × 10^4^ (±1.5 × 10^4^) pulmonary CFU per animal. Although BCG and rBCG-Mkan85B vaccination both reduced the pulmonary CFU (3.3 × 10^3^ ± 0.9 × 10^3^, and 5.0 × 10^3^ ± 1.4 × 10^3^, respectively) (*p* < 0.01) (Figure 1), the efficacy of rBCG-Mkan85B was comparable to that of BCG. In the lungs of the unvaccinated mice, large granuloma nodules were predominant and consisted of epithelioid cells (Figure 2A,D). Acid-fast bacilli were detected in the granulomas using Ziehl-Neelsen staining (Figure 2G,J). Although granuloma nodules were also observed in the lungs of the BCG- or rBCG-Mkan85B-vaccinated animals (Figure 2B,C), the vaccination with BCG or rBCG-Mkan85B reduced granuloma nodule formation within the lungs, and few acid-fast bacilli were detected in the granulomas using Ziehl-Neelsen staining (Figure 2H,I).

### 3.2. rBCG-Mkan85B/DNA-Mkan85B Prime–Boost Immunization Elicits Protection against Intratracheal Infection with M. kansasii

**Figure 3:**
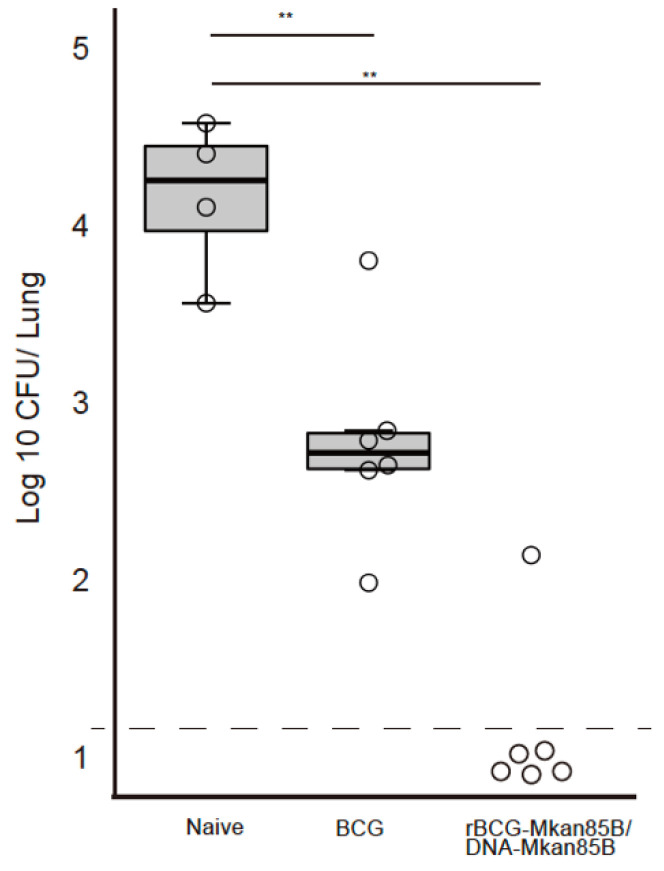
rBCG-Mkan85B/DNA-Mkan85B vaccination protects against M. kansasii infection. The CB6F1 mice were either left unimmunized or immunized with BCG or rBCG-Mkan85B/DNA-Mkan85B and then challenged with a virulent M. kansasii strain for another 6 weeks. The immunization schedule is shown in Figure S1B. The mice were sacrificed at 6 weeks after M. kansasii infection. The number of M. kansasii in individual mouse lungs was analyzed using the colony assay method. The data represent two independent experiments with five to six mice per group. The detection limit for bacilli in the tissue homogenate was 15 CFU. The error bars represent the SD. ** *p* < 0.01.

Next, we evaluated the efficacy of the rBCG-Mkan85B/DNA-Mkan85B prime–boost vaccination against *M.* *kansasii* infection. The CB6F1 mice were either left unimmunized or immunized with BCG or rBCG-Mkan85B/DNA-Mkan85B and then challenged with a virulent *M.* *kansasii* strain for another 6 weeks. The immunization schedule is shown in Figure S1B. As shown in Figure 3, the rBCG-Mkan85B/DNA-Mkan85B vaccination drastically reduced the bacterial burden (Figure 3). Live bacteria were not detected in the lungs of the rBCG-Mkan85B/DNA-Mkan85B-vaccinated CB6F1 mice except for one mouse (Figure 3).

### 3.3. rBCG-Mkan85B/DNA-Mkan85B Prime–Boost Vaccination Induced Polyfunctional CD8^+^ T Cells Specific for the Major Secretory Protein Ag85B in the Lung and Spleen

To analyze the adaptive immune responses induced by BCG, rBCG-Mkan85B, and rBCG-Mkan85B/DNA-Mkan85B vaccination, we examined the induction of antigen-specific polyfunctional T cells using an intracellular cytokine staining (ICS) method.

**Figure 4:**
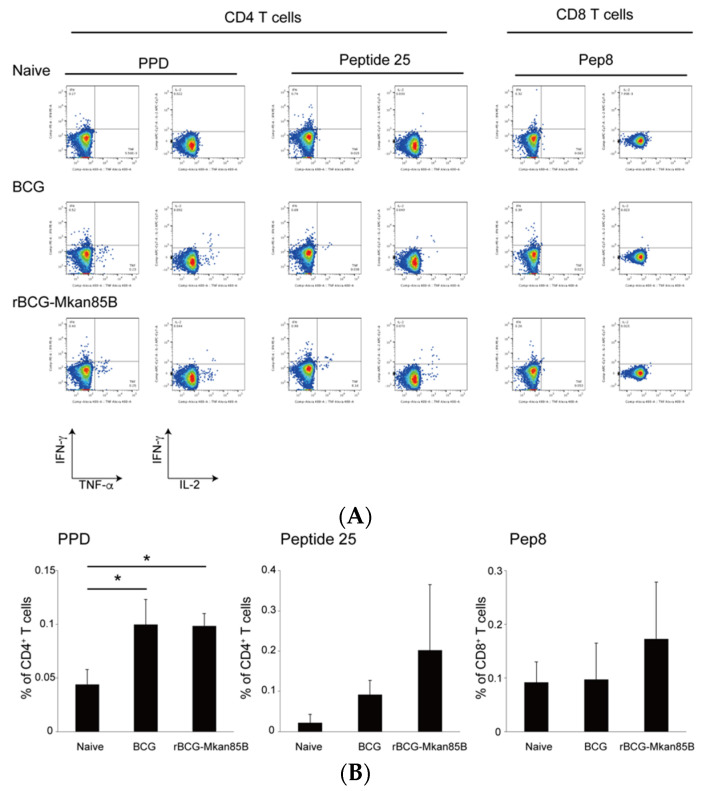
*M.kansasii*-specific polyfunctional CD4^+^ and CD8^+^ T cells in BCG- or rBCG-Mkan85B-vaccinated CB6F1 mice. (**A**) A representative fluorogram of epitope-specific polyfunctional CD4^+^ and CD8^+^ T cell inductions in splenocytes from unvaccinated and vaccinated mice infected with *M.* *kansasii*; TNF is shown on the *x*-axis and IFN-γ is shown on the *y*-axis. (**B**) Polyfunctional CD4^+^ and CD8^+^ T cells from unvaccinated and vaccinated CB6F1 mice infected with *M.* *kansasii*. Polyfunctional CD4^+^ T cell induction by stimulation with PPD (left panel), polyfunctional CD4^+^ T cell induction by stimulation with peptide 25 (middle panel), and polyfunctional CD8^+^ T cell induction by stimulation with Pep8 (right panel) in unvaccinated, BCG-, and rBCG-Mkan85B-vaccinated mice are shown. The data represent two independent experiments with three to four mice per group. The error bars represent the SD. * *p* < 0.05.

Splenocytes were obtained from each mouse vaccinated with BCG or rBCG-Mkan85B at six weeks after *M. kansasii* infection (Figure S1A). The cells were stimulated with 9-mer Ag85B CD8 epitope peptides (Pep8) [13], the 15-mer Ag85B CD4 epitope peptide (peptide 25) [18], or PPD (2 µg/mL) in vitro. The Pep8 and peptide 25 were modified to match the amino acid sequence of *M. kansasii*. Polyfunctional T cells, defined as those that produced two or three cytokines, were analyzed using flow cytometry. The BCG and rBCG-Mkan85B vaccination elicited similar levels of PPD-specific polyfunctional CD4^+^ T cells (Figure 4A,B). The rBCG-Mkan85B vaccination induced higher levels of peptide 25-specific polyfunctional CD4^+^ T cells than the BCG vaccination, but the difference was not significant (Figure 4A,B). On the other hand, the BCG or rBCG-Mkan85B vaccination alone could not induce *M.* *kansasii*-specific CD8^+^ T cells (Figure 4A,B). These results were consistent with our previous reports showing that BCG immunization could not induce polyfunctional CD8^+^ T cells specific to M. tuberculosis Ag85B [13].

**Figure 5:**
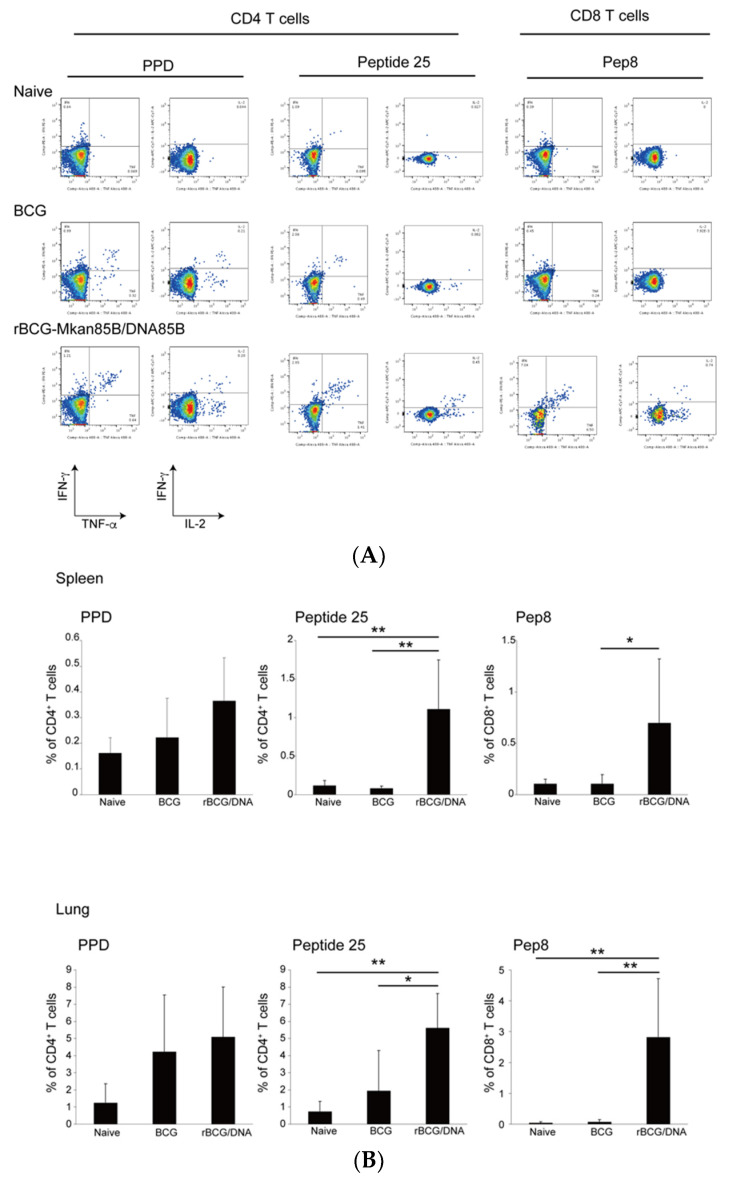
*M.kansasii*-specific polyfunctional CD4^+^ and CD8^+^ T cells in rBCG-Mkan85B/DNA-Mkan85B-prime–boost-vaccinated CB6F1 mice. (**A**) A representative fluorogram of epitope-specific polyfunctional CD4^+^ and CD8^+^ T cell inductions in splenocytes from unvaccinated and vaccinated CB6F1 mice infected with *M.* *kansasii*; TNF is shown on the *x*-axis and IFN-γ is shown on the *y*-axis. (**B**) Polyfunctional CD4^+^ and CD8^+^ T cells from the unvaccinated and vaccinated CB6F1 mice infected with *M.* *kansasii*. Polyfunctional CD4^+^ T cell induction by stimulation with PPD (left panel), polyfunctional CD4+ T cell induction by stimulation with peptide 25 (middle panel), and polyfunctional CD8^+^ T cell induction by stimulation with Pep8 (right panel) in unvaccinated, BCG-, and rBCG-Mkan85B/DNA-Mkan85B-vaccinated mice are shown. The data represent two independent experiments with five to six mice per group. The error bars represent the SD. * *p* < 0.05. ** *p* < 0.01.

Next, we investigated the immune responses induced by rBCG-Mkan85B/DNA-Mkan85B prime–boost vaccination using the same method. In addition to splenocytes, immune cells isolated from the lungs were also analyzed. As shown in Figure 5, the rBCG-Mkan85B/DNA-Mkan85B immunization induced both *M.* *kansasii*-specific polyfunctional CD4^+^ and CD8^+^ T cells (Figure 5). Interestingly, *M.* *kansasii*-specific polyfunctional CD4^+^ and CD8^+^ T cells were more strongly induced in the lung than in the spleen by the rBCG-Mkan85B immunization followed by the DNA-Mkan85B boost (Figure 5A,B).

## 4. Discussion

Cellular immunity plays an important role in controlling infections by intracellular pathogens, such as *M. tuberculosis*, NTM, and viruses. Previous studies by us and other researchers have shown that BCG immunization only weakly activates CD8^+^ T cells, in contrast to its robust activation of CD4^+^ T cells [13,22,23]. We previously suggested that rBCG-prime and DNA-boost strategies could induce CD8^+^ T cell immunity to Ag85B of the *M. tuberculosis* [13] and HIV envelope proteins [17,24]. In this study, we observed that the rBCG-Mkan85B/DNA-Mkan85B prime–boost scheme induced antigen-specific polyfunctional CD8^+^ T cells, which produced double or triple combinations of IFN-γ, IL-2, and TNF in addition to polyfunctional CD4^+^ T cells.

Generally, NTM patients are considered to have been infected by environmental NTM, not by NTM from other patients. In other words, NTM do not cause airborne or droplet infections, unlike TB and SARS-CoV-2, the latter of which is responsible for coronavirus disease 2019 (COVID-19). However, increases in NTM incidence rates have been reported recently [1,2]. Moreover, the emergence of multidrug-resistant NTM is a serious clinical concern, and vaccines for NTM are not available currently. BCG has been used as a TB vaccine, and some researchers have reported that BCG vaccination can reduce the risk of NTM infection, such as infection with *M. avium-intracellulare* complex (MAC) [19,20,21], *M. malmoense*, *M. marinum,* or *M. kansasii* [20]. Abate et al. reported that BCG vaccination induced *M. avium* and *M. abscessus* cross-reactive T cells in humans [11]. Previously, we reported that various CD8^+^ T cell epitopes of the mycobacterial Ag85 complex elicited similar levels of cytokine production by polyfunctional T cells in mice vaccinated with an rBCG-Mkan85B/DNA-Mkan85B prime–boost strategy [13].

In the present study, we first challenged unvaccinated and BCG- or rBCG-Mkan85B-vaccinated mice with virulent *M. kansasii*. We showed that both BCG and rBCG-Mkan85B could protect the mice from virulent *M. kansasii* infection. However, contrary to our expectation, the efficacy of rBCG-Mkan85B vaccination was not superior to that of BCG vaccination. An ICS analysis revealed that *M. kansasii* antigen-specific polyfunctional CD4^+^ T cells were equally induced by BCG and rBCG-Mkan85B vaccination. However, similar to previous reports [13,22,23,25], we observed that BCG immunization and in vitro restimulation with *M. kansasii* peptides could not induce antigen-specific polyfunctional CD8^+^ T cells. Moreover, the present study demonstrated that rBCG-Mkan85B vaccination alone was not sufficient to induce *M. kansasii* Ag85B-specific polyfunctional CD8^+^ T cells. Therefore, we immunized mice with rBCG-Mkan85B and boosted them with DNA-Mkan85B three times. BCG and recombinant BCG prime–boost immunization were reported to be beneficial strategies for inducing antigen-specific CD8^+^ T cells [13,17,24]. In the present study, the bacterial burden in the lungs was remarkably reduced in the mice vaccinated with the rBCG-Mkan85B/DNA-Mkan85B prime–boost strategy. In addition, we revealed that rBCG-Mkan85B/DNA-Mkan85B vaccination could induce antigen-specific polyfunctional CD8^+^ and CD4^+^ T cells. Regarding antigen-specific CD4^+^ T cells, several critical roles for eliminating mycobacteria have been reported [11,26,27]. In fact, AIDS patients with low CD4^+^ T cell counts have a high risk of developing NTM infection. In addition to that of CD4^+^ T cells, the importance of CD8^+^ T cells to protecting against mycobacterial infection was revealed by previous studies by other researchers. For instance, the airborne infection of mice [28], calves [29], and rhesus macaques [27] with *M. avium* induced the activation of both CD4^+^ T cells and CD8^+^ T cells. However, the significance of CD8^+^ T cells in protection against NTM infection, especially *M. avium* infection, has been controversial. Although the depletion or genetic elimination of CD8^+^ T cells significantly decreases immunological responses to TB and increases susceptibility to *M. tuberculosis* [30,31], the depletion of CD8^+^ T cells was reported to be unrelated to *M. avium* infection [32]. Gilbertson et al. suggested that the bystander activation of CD8^+^ T cells occurred during experimental *M. avium* infection [28]. On the other hand, the role of CD8^+^ T cells in *M. kansasii* infection has not been investigated thoroughly. In the present study, although BCG or rBCG-Mkan85B immunization could activate only *M. kansasii*-specific polyfunctional CD4^+^ T cells, rBCG-Mkan85B/DNA-Mkan85B prime-boost vaccination was able to elicit *M. kansasii*-specific polyfunctional CD8^+^ T cells. Nevertheless, the BCG- or rBCG-Mkan85B-immunized mice were shown to be protected against *M. kansasii* infection, and the rBCG-Mkan85B/DNA-Mkan85B prime-boost strategy drastically reduced the bacterial burden in the lungs of the mice. The results suggest a role for antigen-specific polyfunctional CD8^+^ T cells in protecting against *M. kansasii* infection.

Since the end of 2019, COVID-19 has been a serious public health concern worldwide. Looking back on history, human beings have suffered from many kinds of infections. We have to continue to fight various pathogenic microorganisms and infectious diseases. Vaccines are the gold standard for protecting against infectious diseases. One of the aims of a vaccine is to induce effective neutralizing antibodies against the target pathogen. To protect against several pathogenic microbes, such as mycobacteria, the activation of cellular immunity is required in addition to the induction of humoral immunity. BCG has been used globally as a live attenuated vaccine against TB for a century. Various recombinant BCG strains have been designed, developed, and evaluated as novel vaccines against various infectious diseases. For instance, several rBCG strains expressing HIV antigens, such as HIV gag and env, have been reported to induce anti-HIV neutralizing antibodies and antigen-specific CD4^+^ and CD8^+^ T cells [24,33,34]. We and others reported that a prime–boost strategy could elicit relatively high levels of immunological activity against pathogens [13,33,35,36,37]. Moreover, BCG has the advantages of providing safety to healthy subjects and potent adjuvant activities. Therefore, rBCG might be a candidate for use in novel vaccines against emerging infectious diseases for which vaccines have not yet been developed.

## 5. Conclusions

In conclusion, we demonstrated that BCG and rBCG-Mkan85B immunization protected CB6F1 mice from *M. kansasii* infection by inducing *M. kansasii*-specific polyfunctional CD4^+^ T cells. Furthermore, rBCG-Mkan85B/DNA-Mkan85B prime–boost vaccination induced antigen-specific CD4^+^ and CD8^+^ T cells and drastically reduced the CFU of *M. kansasii* in the lungs of vaccinated mice. Our data suggest that the combination of rBCG expressing Ag85B derived from *M. kansasii* with DNA that also expresses Ag85B derived from *M. kansasii* may be effective in enhancing antigen-specific T cells, resulting in more efficient control of *M. kansasii* infection.

## Data Availability

The authors declare that the data supporting the findings of this study are available within the paper.

## Supplementary Materials

The following are available online at https://www.mdpi.com/article/10.3390/vaccines9111260/s1, Figure S1: Immunization Schedule, Figure S2: Gating tree for functional characterization of distinct populations of responding CD4^+^ or CD8^+^ T cells using polychromatic flow cytometry.
