# Supplementary material for: Recombinant BCG-Prime and DNA-Boost Immunization Confers Mice with Enhanced Protection against Mycobacterium kansasii
Source: Vaccines (Basel). 2021 Nov 1;9(11):1260. doi: 10.3390/vaccines9111260 (PMC8618695; doi:10.3390/vaccines9111260)
Supplement: Supplementary file 1 [file vaccines-09-01260-s001.zip › vaccines-1436974-supplementary.pdf]

Supplent Fig. S1

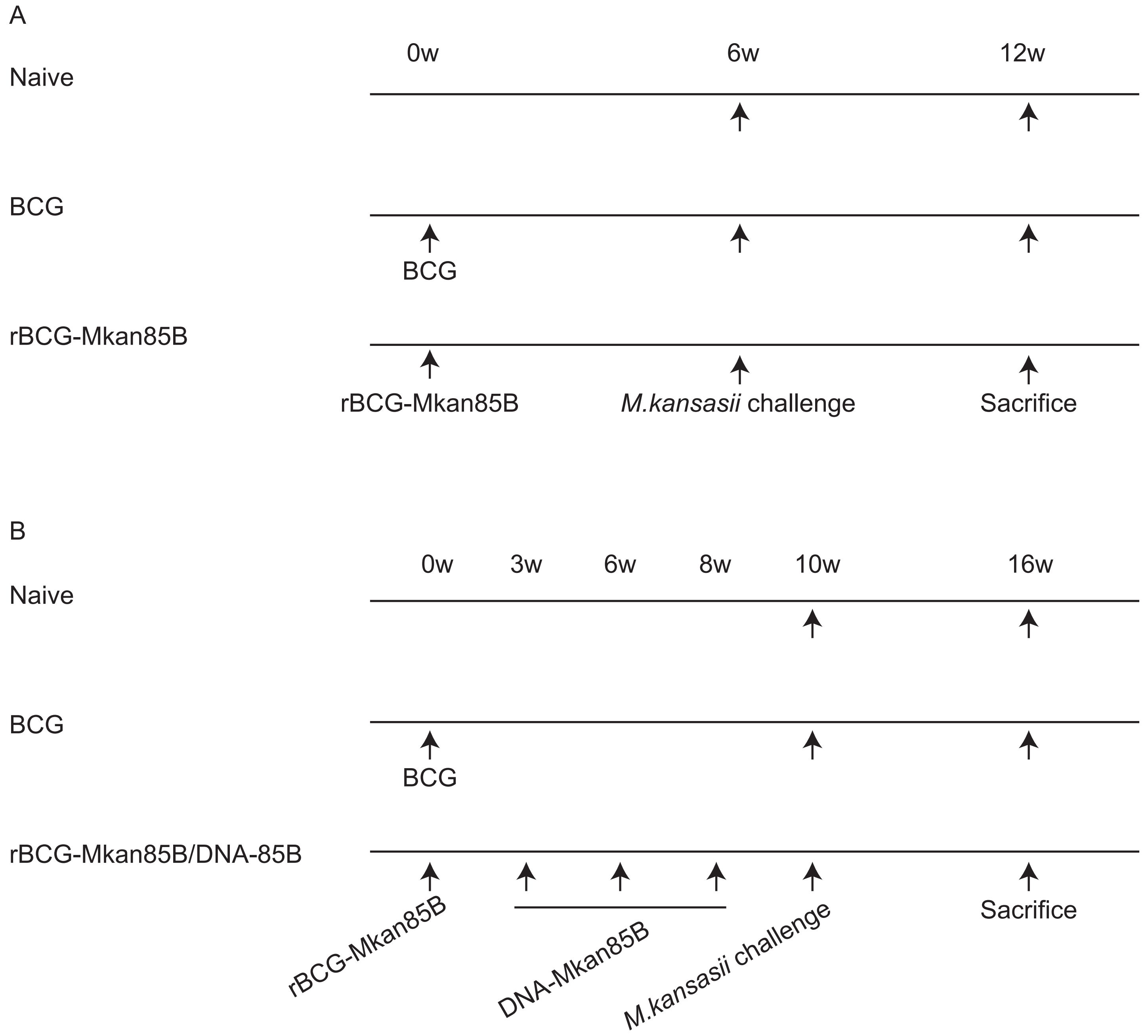


Supplemental Figure S1. Immunization schedule.

(A) CB6F1 (H2b/d) mice were immunized with the BCG vaccine or rBCG-Mkan85B at a concentration of 4 ×106 CFU or 0.1 mg of bacilli i.d. for 6 weeks, followed by nasal exposure to virulent *M. kansasii* strain infection for another 6 weeks. (B) CB6F1 (H2b/d) mice were immunized with the BCG vaccine or rBCG-Mkan85B at a concentration of 4 ×106 CFU or 0.1 mg of bacilli i.d. and 100 μg of plasmid DNA in saline i.m. three times. Two weeks after the final DNA-Mkan85B immunization, the mice were infected with *M. kansasii*.

Supplent Fig. S2

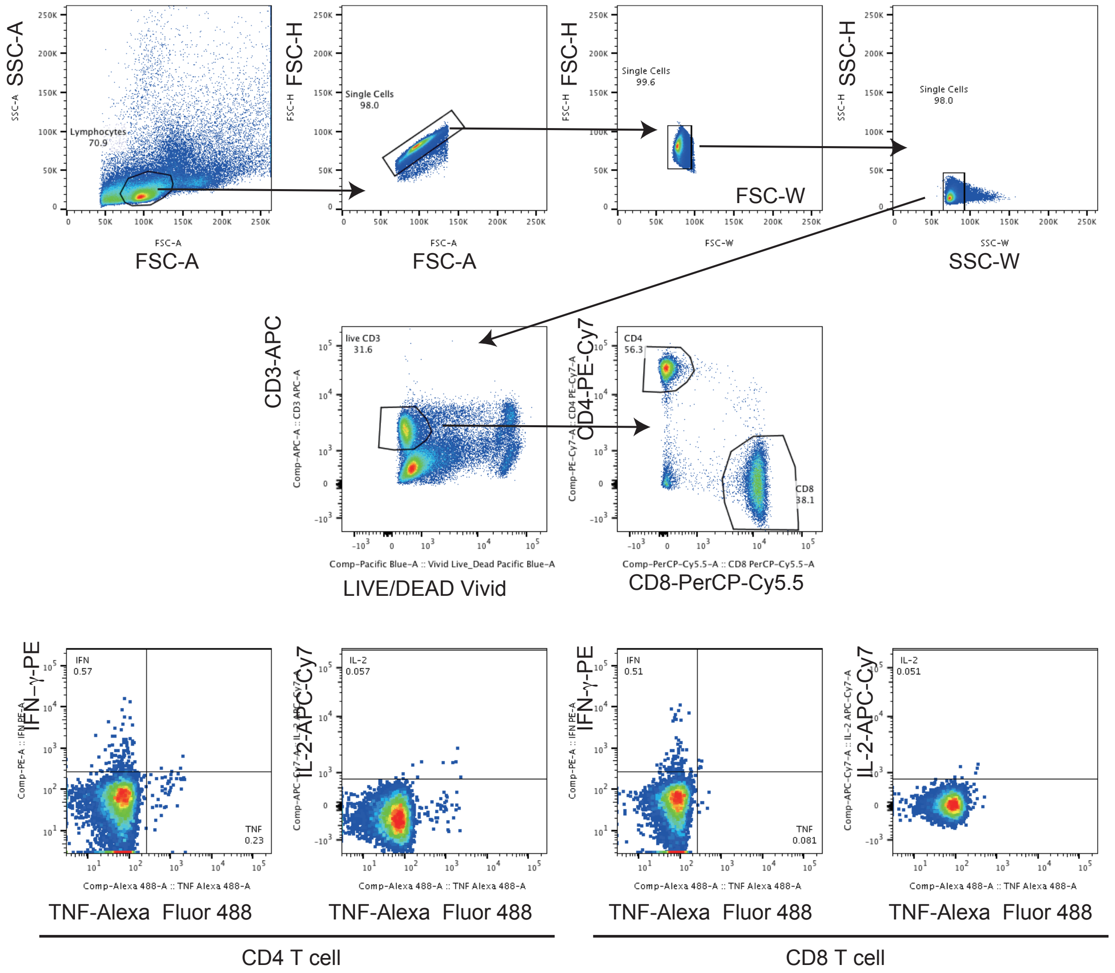


Supplemental Figure S2. Gating tree for functional characterization of distinct populations of responding CD4⁺ or CD8⁺ T cells using polychromatic flow cytometry

The gating strategy used to identify IFN-γ-, IL-2- and TNF-producing CD4⁺ and CD8⁺ T cells in splenocytes from a representative mouse is shown. The upper 4 panels show the initial gating of total events, including a singlet cell gate, followed by selection for lymphocytes. Live CD3⁺ T cells were identified as LIVE/DEAD ViViD-CD3⁺ cells. CD8⁺ and CD4⁺ T cells were further identified by CD8 and CD4 expression patterns. Antigen-specific IFN-γ-, IL-2- and TNF-producing CD4⁺ T cells or CD8⁺ T cells were gated as shown. The cells producing three, any two and any one cytokine were determined by Boolean combinations. The sum of the three and two cytokine-producing cells was measured as the polyfunctional T cells specific for PPD or the epitope peptides.
